# Mucin-Like Domain of Mucosal Addressin Cell Adhesion Molecule-1 Facilitates Integrin α4β7-Mediated Cell Adhesion Through Electrostatic Repulsion

**DOI:** 10.3389/fcell.2020.603148

**Published:** 2020-12-14

**Authors:** MengYa Yuan, YanRong Yang, Yue Li, ZhanJun Yan, ChangDong Lin, JianFeng Chen

**Affiliations:** ^1^State Key Laboratory of Cell Biology, Shanghai Institute of Biochemistry and Cell Biology, Center for Excellence in Molecular Cell Science, Chinese Academy of Sciences, University of Chinese Academy of Sciences, Shanghai, China; ^2^School of Life Science, Hangzhou Institute for Advanced Study, University of Chinese Academy of Sciences, Hangzhou, China; ^3^Suzhou Ninth People’s Hospital, Soochow University, Suzhou, China

**Keywords:** MAdCAM-1, integrin α4β7, mucin-like domain, electrostatic repulsion, cell adhesion

## Abstract

The homing of lymphocytes from blood to gut-associated lymphoid tissue is regulated by interaction between integrin α4β7 with mucosal vascular addressin cell adhesion molecule 1 (MAdCAM-1) expressed on the endothelium of high endothelial venules (HEVs). However, the molecular basis of mucin-like domain, a specific structure of MAdCAM-1 regulating integrin α4β7-mediated cell adhesion remains obscure. In this study, we used heparan sulfate (HS), which is a highly acidic linear polysaccharide with a highly variable structure, to mimic the negative charges of the extracellular microenvironment and detected the adhesive behaviors of integrin α4β7 expressing 293T cells to immobilized MAdCAM-1 *in vitro*. The results showed that HS on the surface significantly promoted integrin α4β7-mediated cell adhesion, decreased the percentage of cells firmly bound and increased the rolling velocities at high wall shear stresses, which was dependent on the mucin-like domain of MAdCAM-1. Moreover, breaking the negative charges of the extracellular microenvironment of CHO-K1 cells expressing MAdCAM-1 with sialidase inhibited cell adhesion and rolling velocity of 293T cells. Mechanistically, electrostatic repulsion between mucin-like domain and negative charges of the extracellular microenvironment led to a more upright conformation of MAdCAM-1, which facilitates integrin α4β7-mediated cell adhesion. Our findings elucidated the important role of the mucin-like domain in regulating integrin α4β7-mediated cell adhesion, which could be applied to modulate lymphocyte homing to lymphoid tissues or inflammatory sites.

## Introduction

Integrins are important cell surface adhesion molecules, which are widely expressed on the cell membrane. They are heterodimers formed by non-covalent bonds between α and β subunits. In vertebrates, 18 α subunits and 8 β subunits combine to form 24 different integrins, specifically or cross-recognizing multiple extracellular matrix ligands (Hynes, 2002; Takada et al., 2007). Based on EM and atomic structures of integrins, the extracellular domain of integrin exists in at least three distinct global conformational states: (i) bent with a closed headpiece, (ii) extended with a closed headpiece, (iii) extended with an open headpiece. The closed and open headpieces of integrin have a low and high affinity for the ligand, respectively (Wang et al., 2018).

The homing of lymphocytes from blood to secondary lymphoid nodes or inflammatory sites is regulated by interaction with specific capillary venules, especially high endothelial venules (HEVs) (Ager, 2017). A highly ordered adhesion cascade mediates the recruitment process, including tethering and rolling of lymphocytes along vessel walls of HEVs, chemokine-induced integrin activation, firm arrest and transendothelial migration (von Andrian and Mempel, 2003; Lin et al., 2019). During this process, α4 integrins (α4β1 and α4β7) and β2 integrins (e.g., αLβ2 and αMβ2) on lymphocytes bind to their distinct ligands on vascular endothelial cells to mediate cell adhesion and migration. Among these integrins, α4 integrins, especially integrin α4β7 mediates both rolling and firm adhesion to mucosal vascular addressin cell adhesion molecule 1 (MAdCAM-1) (Berlin et al., 1993; Sun et al., 2014; Wang et al., 2018), which plays an important role to support efficient lymphocyte homing.

MAdCAM-1 is the primary ligand of integrin α4β7, specifically expressed on the endothelium of HEVs in the gut and gut-associated lymphoid tissues such as Peyer’s patches (PPs) and mesenteric lymph nodes (MLNs) (Springer, 1994; Cox et al., 2010). Integrin α4β7-MAdCAM-1 binding-mediated pathological lymphocyte recruitment to the gut initiates and accelerates inflammatory bowel disease (IBD), consisting of ulcerative colitis (UC) and Crohn’s disease (CD) (Hoshino et al., 2011). Thus, the regulation of lymphocyte adhesion mediated by the interaction between integrin α4β7 and MAdCAM-1 need to be further illustrated. MAdCAM-1 is a type I transmembrane glycoprotein molecule and belongs to a subclass of the immunoglobulin superfamily (IgSF), containing two Ig-like domains and a mucin-like domain (Tan et al., 1998). Mucin-like domain is a serine/threonine-rich region which serves as a backbone to support the interaction with lymphocytes (Briskin et al., 1993). Although the crystal structure of MAdCAM-1 has revealed that MAdCAM-1 binds directly to integrin α4β7 via Asp42 in the CD loop of Ig-like domain 1 (D1), and an unusual long D strand in Ig-like domain 2 (Ig-like domain 2) is also necessary for α4β7 binding (Tan et al., 1998), the function and molecular basis of mucin-like domain on the regulation of integrin α4β7-mediated cell adhesion is poorly understood. Considering the extracellular parts of plasma membrane proteins are generally glycosylated, which gives the microenvironment of plasma membrane negative charges, whether mucin-like domain forms electrostatic repulsion with the extracellular microenvironment, so as to affect integrin α4β7-mediated cell adhesion remains obscure.

In this study, we used heparan sulfate (HS) to mimic the negative charges of the extracellular microenvironment and detected the adhesive behaviors of integrin α4β7 expressing 293T cells to immobilized MAdCAM-1 *in vitro*. The results showed that HS on the surface promoted integrin α4β7-mediated cell adhesion to immobilized MAdCAM-1, which was dependent on the mucin-like domain of MAdCAM-1. Moreover, breaking the negative charges of the extracellular microenvironment of CHO-K1 cells expressing MAdCAM-1 with sialidase inhibited cell adhesion and rolling velocity of 293T cells. Mechanistically, electrostatic repulsion between mucin-like domain and negative charges of the extracellular microenvironment led to a more upright conformation of MAdCAM-1, which facilitates integrin α4β7-mediated cell adhesion. Our findings elucidated the important role of the mucin-like domain in regulating integrin α4β7-mediated cell adhesion, which could be applied to modulate lymphocyte homing to lymphoid tissues or inflammatory sites.

## Results

### Negative Charges on the Surface Facilitates Integrin α4β7-Mediated Cell Adhesion to Immobilized MAdCAM-1

**FIGURE 1:**
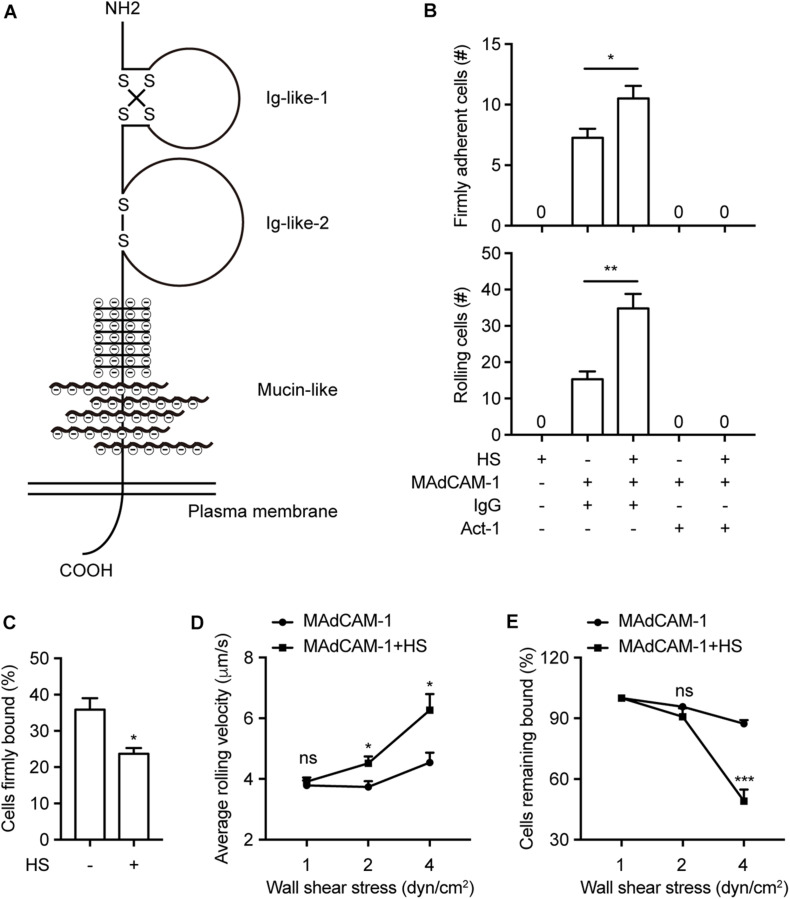
HS on the surface promotes integrin α4β7-mediated cell adhesion to immobilized MAdCAM-1. **(A)** Schematic diagram of the structure of MAdCAM-1, the extracellular domain of MAdCAM-1 includes Ig-like-1, Ig-like-2 and mucin-like (serine/threonine-rich) three domains. **(B–E)** Adhesive behaviors of 293T-α4β7 cells on immobilized HS (100 μg/ml) or MAdCAM-1 (25 μg/ml) substrates in 1 mM Ca^2+^/Mg^2+^. The number of rolling and firmly adherent 293T-α4β7 cells was measured at a wall shear stress of 1 dyn/cm^2^. Cells were pre-treated with murine IgG (10 μg/ml) or α4β7 blocking antibody Act-1 (10 μg/ml) for 10 min at 37°C **(B)**. Percentage of cells firmly bound to ligand at a wall shear stress of 1 dyn/cm^2^ **(C)**. Average rolling velocity of 293T-α4β7 cells that adhered to MAdCAM-1 substrates at indicated wall shear stresses **(D)**. Resistance of 293T-α4β7 cells to detachment at increasing wall shear stresses. The total number of cells remaining bound at each indicated wall shear stress was determined as a percent of adherent cells at 1 dyn/cm^2^ **(E)**. One representative result of three independent experiments is shown in **(B–E)**. Data represent the mean ± SEM (*n* ≥ 3) in **(B–E)**. *p < 0.05, **p < 0.01, ***p < 0.001, ns: not significant (Student’s *t*-test).

To study whether negative charges of the extracellular microenvironment affect integrin α4β7-mediated cell adhesion to MAdCAM-1 *in vitro*, we firstly purified Fc/His tagged extracellular domain of MAdCAM-1 (including Ig-like-1, Ig-like-2 and mucin-like domains, Met1-Gln317) in 293T cells (Figure 1A). Then we examined the effect of negative charges on cell adhesion to immobilized MAdCAM-1 in the presence of physiological cations (1 mM Ca^2+^ + Mg^2+^). Heparan sulfate (HS) was used to mimic the extracellular microenvironment, which is a highly acidic linear polysaccharide with a very variable structure (Simon Davis and Parish, 2013). It is widely expressed on cell surfaces and the presence of sulfate groups at specific positions in HS chains imparts an overall high negative charge (Esko and Lindahl, 2001; Weiss et al., 2017). Firstly, we studied the adhesive behavior of 293T cells stably expressing integrin α4β7 (293T-α4β7) on MAdCAM-1 with HS or not in shear flow using flow chamber system. The shear stress was increased incrementally and the velocity of rolling cells at each increment was determined. At the wall shear stress of 1 dyn/cm^2^, HS coated on the surface significantly increased the numbers of both firmly adherent cells (7.25 ± 0.75 vs. 10.5 ± 1.04) and rolling cells (15.25 ± 2.18 vs. 34.75 ± 4.03) (Figure 1B and Supplementary Movies S1–S5). Meanwhile, cells did not directly adhere to HS at all and cells pre-treated with α4β7 blocking antibody Act-1 did not adhere to MAdCAM-1 (Figure 1B), indicating that the adhesion is specifically integrin α4β7 dependent. The percentage of cells firmly bound to ligand decreased from 35.86 to 23.70% by the addition of HS (Figure 1C), which means HS might prefer to promote rolling adhesion. Consistent to the hypothesis, the average rolling velocity of cells was upregulated at the wall shear stress of 2 dyn/cm^2^ and 4 dyn/cm^2^ (Figure 1D). Furthermore, we tested the effect of HS on the strength of α4β7-mediated adhesion to MAdCAM-1 by calculating the cell resistance to detachment by increasing wall shear stresses. Cells adhered to the surface with HS detached much more rapidly from MAdCAM-1 (Figure 1E). These data demonstrate that HS on the surface promotes integrin α4β7-mediated cell adhesion to immobilized MAdCAM-1, which might be due to the negative charges of sulfate groups in HS chains.

### Mucin-Like Domain of MAdCAM-1 Is Responsible for HS-Enhanced Integrin α4β7-Mediated Cell Adhesion

**FIGURE 2:**
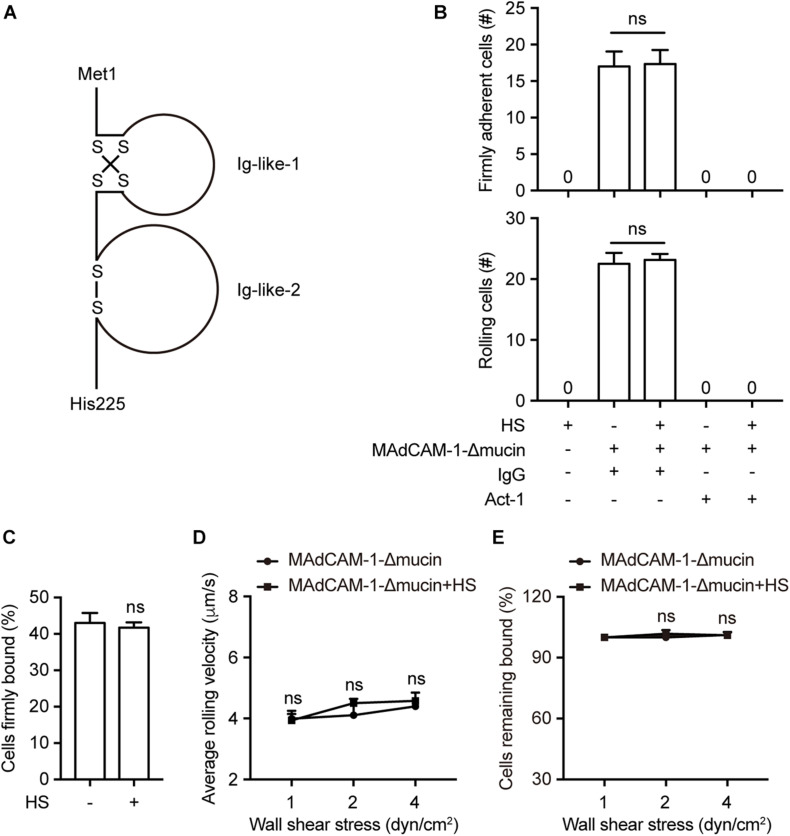
HS-enhanced integrin α4β7-mediated cell adhesion is dependent on mucin-like domain of MAdCAM-1. **(A)** Schematic diagram of the structure of the extracellular domain of MAdCAM-1 with a deletion of mucin-like domain. **(B–E)** Adhesive behaviors of 293T-α4β7 cells on immobilized HS (100 μg/ml) or MAdCAM-1-Δmucin (100 μg/ml) substrates in 1 mM Ca^2+^/Mg^2+^. The number of rolling and firmly adherent 293T-α4β7 cells was measured at a wall shear stress of 1 dyn/cm^2^. Cells were pre-treated with murine IgG (10 μg/ml) or α4β7 blocking antibody Act-1 (10 μg/ml) for 10 min at 37°C **(B)**. Percentage of cells firmly bound to ligand at a wall shear stress of 1 dyn/cm^2^ **(C)**. Average rolling velocity of 293T-α4β7 cells that adhered to MAdCAM-1-Δmucin substrates at indicated wall shear stresses **(D)**. Resistance of 293T-α4β7 cells to detachment at increasing wall shear stresses **(E)**. One representative result of three independent experiments is shown in **(B–E)**. Data represent the mean ± SEM (*n* ≥ 3) in **(B–E)**. ns: not significant (Student’s *t*-test).

Mucin-like domain contains a serine/threonine-rich region, which may form an electrostatic repulsion with negative charges of the extracellular microenvironment. To investigate whether this specific domain play a role in supporting the interaction with lymphocytes, we deleted the mucin-like domain from the full length MAdCAM-1 (MAdCAM-1-Δmucin). Fc/His tagged extracellular domain of MAdCAM-1-Δmucin (including only Ig-like-1 and Ig-like-2, Met1-His225) (Figure 2A) was also purified in 293T cells. Then we examined the adhesive behavior of 293T-α4β7 cells to immobilized MAdCAM-1-Δmucin with HS or not. As expected, HS coated on the surface could not influence the number and the percentage of either firmly adherent cells (17.00 ± 2.05 vs. 17.33 ± 1.93) or rolling cells (22.50 ± 1.80 vs. 23.17 ± 0.98) at the wall shear stress of 1 dyn/cm^2^ (Figures 2B,C). Similarly, the average rolling velocities of cells adhered to MAdCAM-1-Δmucin with HS or not showed to be comparable at all indicated wall shear stresses (Figure 2D) and the cell resistance to detachment from MAdCAM-1-Δmucin was also not changed by the addition of HS on the surface (Figure 2E). Taken together, HS-enhanced integrin α4β7-mediated cell adhesion is dependent on mucin-like domain of MAdCAM-1.

### Breaking the Negative Charges of the Extracellular Microenvironment Suppresses Integrin α4β7-Mediated Cell Adhesion

**FIGURE 3:**
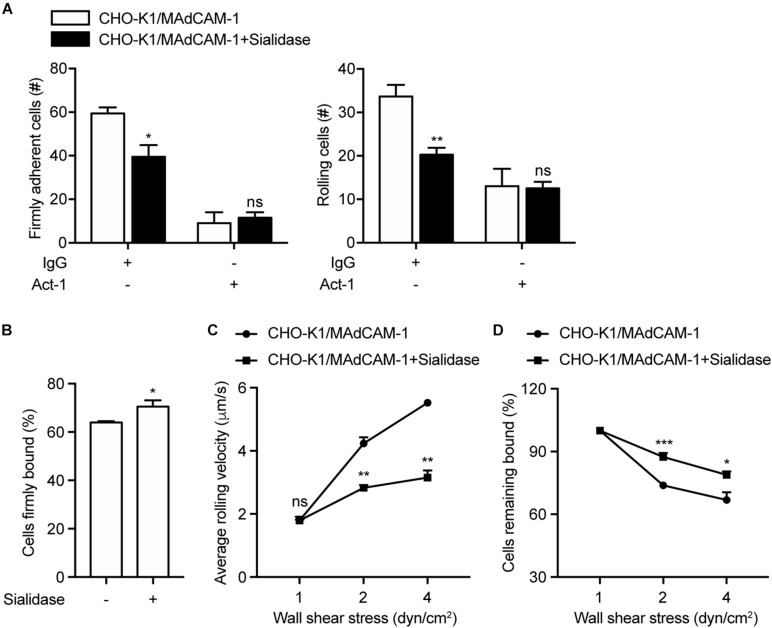
Breaking the negative charges of the extracellular microenvironment suppresses integrin α4β7-mediated cell adhesion. Adhesive behaviors of 293T-α4β7 cells on CHO-K1/MAdCAM-1 cells. **(A)** The number of rolling and firmly adherent 293T-α4β7 cells was measured at a wall shear stress of 1 dyn/cm^2^. Cells were pre-treated with murine IgG (10 μg/ml) or α4β7 blocking antibody Act-1 (10 μg/ml) for 10 min at 37°C. **(B)** Percentage of cells firmly bound to CHO-K1/MAdCAM-1 cells at a wall shear stress of 1 dyn/cm^2^. **(C)** Average rolling velocity of 293T-α4β7 cells that adhered to CHO-K1/MAdCAM-1 cells at indicated wall shear stresses. **(D)** Resistance of 293T-α4β7 cells to detachment at increasing wall shear stresses. One representative result of three independent experiments is shown. Data represent the mean ± SEM (*n* ≥ 3). *p < 0.05, **p < 0.01, ***p < 0.001, ns: not significant (Student’s *t*-test).

To assess the requirement of negative charges of the extracellular microenvironment for integrin α4β7-mediated cell adhesion, CHO-K1 cells stably expressing full length MAdCAM-1 (CHO-K1/MAdCAM-1) were established (Supplementary Figure S1). Sialidase (also known as neuraminidase) was used to remove cell surface anionic sialic acid of glycoconjugate, so as to break the negative charges of the extracellular microenvironment (Thaysen-Andersen et al., 2013; Ashdown et al., 2020). CHO-K1/MAdCAM-1 cells were pre-treated with sialidase or not and then used in flow chamber assay to study the adhesive behavior of 293T-α4β7 cells in shear flow. At the wall shear stress of 1 dyn/cm^2^, the numbers of both firmly adherent cells (59.33 ± 2.85 vs. 39.50 ± 5.32) and rolling cells (33.67 ± 2.67 vs. 20.25 ± 1.60) decreased significantly when CHO-K1/MAdCAM-1 cells were pre-treated with sialidase (Figure 3A). Of note, although 293T-α4β7 cells were pre-treated with α4β7 blocking antibody Act-1, there existed some cells adhered to the surface of CHO-K1/MAdCAM-1 cells (Figure 3A), suggesting that the remaining cell adhesion is integrin α4β7 independent. Meanwhile, pre-treatment with sialidase upregulated the percentage of cells firmly bound to CHO-K1/MAdCAM-1 cells from 64.00 to 70.52% (Figure 3B) and decreased the average rolling velocity of 293T-α4β7 cells at the wall shear stress of 2 and 4 dyn/cm^2^ (Figure 3C). Furthermore, 293T-α4β7 cells showed to be more resistant to detachment by increasing wall shear stresses when adhered to the surface of CHO-K1/MAdCAM-1 cells pre-treated with sialidase (Figure 3D). These data indicate that breaking the negative charges of the extracellular microenvironment by sialidase suppresses integrin α4β7-mediated cell adhesion to MAdCAM-1 expressed on CHO-K1 cells.

### Deletion of Mucin-Like Domain of MAdCAM-1 Abolishes the Influence of Sialidase on Integrin α4β7-Mediated Cell Adhesion to CHO-K1 Cells

**FIGURE 4:**
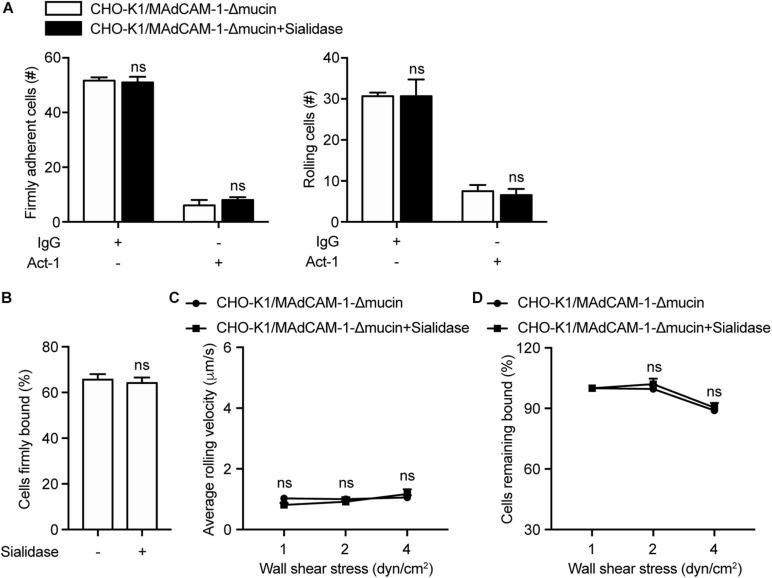
Deletion of mucin-like domain of MAdCAM-1 abolishes the influence of sialidase on integrin α4β7-mediated cell adhesion to CHO-K1 cells. Adhesive behaviors of 293T-α4β7 cells on CHO-K1/MAdCAM-1-Δmucin cells. **(A)** The number of rolling and firmly adherent 293T-α4β7 cells was measured at a wall shear stress of 1 dyn/cm^2^. Cells were pre-treated with murine IgG (10 μg/ml) or α4β7 blocking antibody Act-1 (10 μg/ml) for 10 min at 37°C. **(B)** Percentage of cells firmly bound to CHO-K1/MAdCAM-1-Δmucin cells at a wall shear stress of 1 dyn/cm^2^. **(C)** Average rolling velocity of 293T-α4β7 cells that adhered to CHO-K1/MAdCAM-1-Δmucin cells at indicated wall shear stresses. **(D)** Resistance of 293T-α4β7 cells to detachment at increasing wall shear stresses. One representative result of three independent experiments is shown. Data represent the mean ± SEM (*n* ≥ 3). ns: not significant (Student’s *t*-test).

Above data have demonstrated that mucin-like domain of MAdCAM-1 is responsible for negative charges-enhanced integrin α4β7-mediated cell adhesion. To further confirm the results, CHO-K1 cells stably expressing MAdCAM-1-Δmucin (CHO-K1/MAdCAM-1-Δmucin) were established, which showed a similar level to that of CHO-K1/MAdCAM-1 cells (Supplementary Figure S1). Then we examined the adhesive behavior of 293T-α4β7 cells on CHO-K1/MAdCAM-1-Δmucin cells. Pre-treatment of sialidase did not influence the number and the percentage of either firmly adherent cells (51.67 ± 1.20 vs. 51.00 ± 2.08) or rolling cells (30.67 ± 0.88 vs. 30.67 ± 4.10) and the average rolling velocity at all wall shear stresses (Figures 4A–C). Furthermore, the cell resistance to detachment from CHO-K1/MAdCAM-1-Δmucin cells was also not changed by the treatment of sialidase (Figure 4D). Thus, mucin-like domain of MAdCAM-1 is responsible for the interaction with negative charges of the extracellular microenvironment, which is derived from cell surface anionic sialic acid of CHO-K1 cells.

### Electrostatic Repulsion Between Mucin-Like Domain and Negative Charges of the Extracellular Microenvironment Affects the Conformation of MAdCAM-1

**FIGURE 5:**
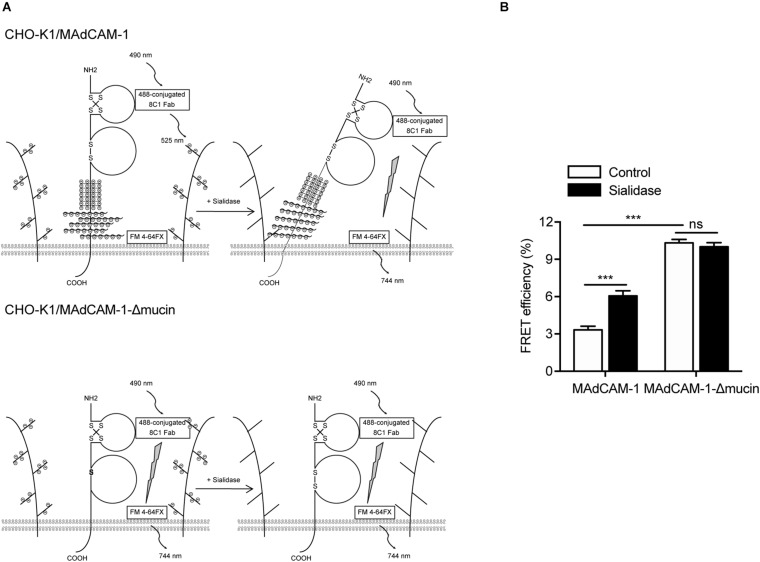
Electrostatic repulsion between mucin-like domain and negative charges of the extracellular microenvironment affects the conformation of MAdCAM-1. **(A)** Experiment setup for measuring FRET efficiency between Ig-like-1 domain and the plasma membrane. A composite of all molecules used is depicted. **(B)** FRET efficiency of CHO-K1/MAdCAM-1 and CHO-K1/MAdCAM-1-Δmucin cells before and after treatment with sialidase. Data represent the mean ± SEM (*n* ≥ 3). ***p < 0.001, ns: not significant (Student’s *t*-test).

Next, we examined the conformational change of MAdCAM-1 ectodomain using fluorescence resonance energy transfer (FRET). To assess the orientation of MAdCAM-1 ectodomain relative to the plasma membrane, Ig-like-1 domain was labeled with Alexa Fluor 488-conjugated 8C1 Fab fragment as the FRET donor. The plasma membrane was labeled with FM 4-64FX as the FRET acceptor (Figure 5A). Pre-treatment with sialidase significantly increased FRET efficiency in CHO-K1/MAdCAM-1 cells (3.33 ± 0.29 vs. 6.05 ± 0.42) but not in CHO-K1/MAdCAM-1-Δmucin cells (10.31 ± 0.28 vs. 10.01 ± 0.33) (Figure 5B), indicating that sialidase-induced bent conformation of MAdCAM-1 ectodomain was dependent on mucin-like domain. Furthermore, the results showed that the FRET efficiency in CHO-K1/MAdCAM-1 cells was much lower than that in CHO-K1/MAdCAM-1-Δmucin cells in the absent of sialidase, which could be due to the fact that deletion of mucin-like domain lowers the height of MAdCAM-1 molecule and Ig-like-1 domain closes to the plasma membrane (Figure 5B). Thus, electrostatic repulsion between mucin-like domain and negative charges of the extracellular microenvironment leads to a more upright conformation of MAdCAM-1, which facilitates integrin α4β7-mediated cell adhesion.

## Discussion

Gut-associated lymphoid tissue (GALT), including isolated and aggregated lymphoid follicles (PPs, MLNs et al.), is one of the largest lymphoid organs in the body (Corr et al., 2008). It contains 70% of the body’s lymphocytes and plays vital roles in gastrointestinal mucosal immunity (Hoshino et al., 2011). The homing of lymphocytes to GALT is dependent of the interaction between integrin α4β7 and MAdCAM-1. The disfunction of integrin α4β7-mediated cell adhesion to MAdCAM-1 could result in pathological lymphocyte recruitment to the gut and the initiation and progression of IBD (Hoshino et al., 2011). In the previous studies, most work focused on the regulation of integrin conformational changes. Chemokine stimulations, inside-out signaling and metal ions all could influence global conformational change of the integrin extracellular domain and the subsequent the adhesin to the distinct ligand (Montresor et al., 2012; Springer and Dustin, 2012; Zhang and Chen, 2012). For example, in Ca^2+^, α4β7 mediates rolling adhesion, whereas in Mg^2+^ alone and in Mn^2+^, α4β7 mediates firm adhesion on MAdCAM-1, mimicking the two steps in lymphocyte accumulation in HEVs or the vasculature at inflammatory sites (de Chateau et al., 2001; Chen et al., 2003). However, little is known whether the conformation of MAdCAM-1 is affected by the extracellular microenvironment.

The crystal structure of MAdCAM-1 exhibits two protruding loops from the two Ig-like domains, the CC’ loop in D1 and DE loop in D2. These two loops are responsible for the interaction between MAdCAM-1 and α4β7 (Sun et al., 2011). Furthermore, a shift in D1 topology from the I2-set to I1-set was demonstrated by new crystals of MAdCAM-1 and two different Fabs, inducing a switch of integrin-binding loop from CC’ to CD (Yu et al., 2013). The different conformations seen in crystal structures suggest that the integrin-binding loop of MAdCAM-1 is inherently flexible, which may explain why MAdCAM-1 could mediate both rolling and firm adhesion by binding to integrin α4β7 in different conformational states.

A mucin-like domain of MAdCAM-1 is unique among integrin ligands, which connects the two Ig-like domains to the plasma membrane. In this respect, MAdCAM-1 resembles selectin ligands (Berg et al., 1993). Selectins selectively mediate rolling adhesion but not firm adhesion on blood vessels and recognize carbohydrate residues displayed on proteins consisting mucin-like regions (Springer, 1994). Furthermore, mucin-like domain is a serine/threonine-rich region. It is proposed that MAdCAM-1 molecule is repelled by the electrostatic repulsion between the highly negatively charged mucin-like region and the extracellular microenvironment, which could help orient the integrin-binding Ig-like domains on cell surface for recognition.

In this study, we found that HS on the surface significantly promoted integrin α4β7-mediated cell rolling adhesion and firm adhesion, decreased the percentage of cells firmly bound and increased the rolling velocities at high wall shear stresses, implying the negative charges of the extracellular microenvironment actually facilitates integrin α4β7-MAdCAM-1 function. Conversely, reducing the negative charges of CHO-K1/MAdCAM-1 extracellular microenvironment by sialidase inhibited cell adhesion and rolling velocity of α4β7 expressing 293T cells. The results of FRET assay gave the direct evidence that electrostatic repulsion between mucin-like domain and negative charges of the extracellular microenvironment leads to a more upright conformation of MAdCAM-1. It is speculated that this electrostatic repulsion-induced extended conformation could further affect the topology of integrin-binding loop in D1 and D2, thereby promoting the binding to integrin α4β7. The detailed conformational change needs to be further clarified with the crystal structures of MAdCAM-1 stabilized by the electrostatic repulsion. Moreover, the deletion of the mucin-like domain significantly increased the FRET efficiency between Ig-like-1 domain and the plasma membrane, indicating a lower height of MAdCAM-1 molecule. Thus, the distance between the binding sites of MAdCAM-1 and integrin α4β7 is also thought to influence the binding efficiency of these two molecules.

**FIGURE 6:**
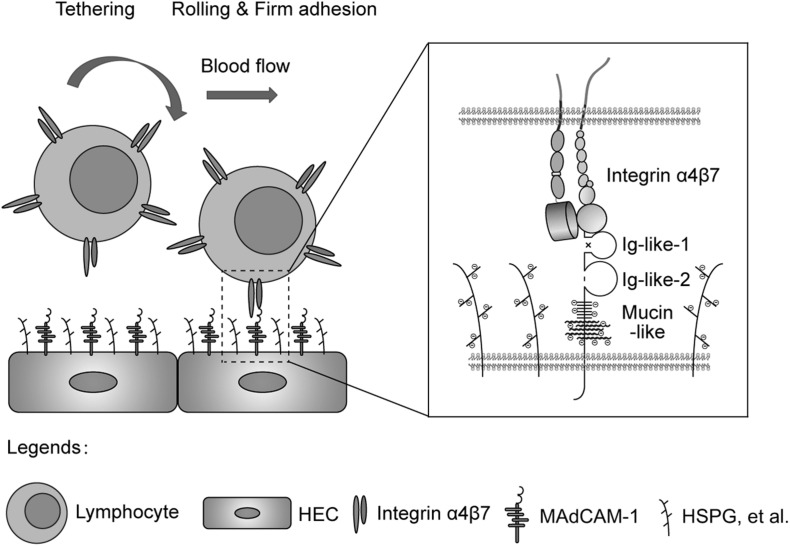
Electrostatic repulsion between mucin-like domain and negative charges of the extracellular microenvironment facilitates integrin α4β7-mediated cell adhesion. Schematic diagram of the adhesion of lymphocytes from blood to the endothelium of HEVs in gut-associated lymphoid tissues. HEC, high endothelial cell; HSPG, heparan sulfate proteoglycan.

In conclusion, mucin-like domain of MAdCAM-1 facilitates integrin α4β7-mediated cell adhesion through electrostatic repulsion with negatively charged extracellular microenvironment (Figure 6), which could be applied to modulate lymphocyte homing to lymphoid tissues or inflammatory sites.

## Materials and Methods

### Protein Expression and Purification

Recombinant human extracellular domain of MAdCAM-1 (Met1-Gln317) or MAdCAM-1-Δmucin (Met1-His225) was constructed in vector pHLsec-Fc/His using restriction enzymes *Age*I and *Kpn*I. 293T cells were transiently transfected with plasmids expressing MAdCAM-1 or MAdCAM-1-Δmucin by the method of Ca_3_(PO_4_)_2_ transfection as described (Chen et al., 2003). Recombinant proteins were purified with Protein A Agarose (Thermo Fisher Scientific).

### Cell Lines

Human embryonic kidney HEK293T cells were cultured at 37°C with 5% CO_2_ in Dulbecco’s modified Eagle medium (DMEM) (Gibco) containing 2 mM L-glutamine, 100 U/mL penicillin, 100 μg/mL streptomycin, and 10% (vol/vol) fetal bovine serum (Gibco). CHO-K1 cells were cultured at 37°C with 5% CO_2_ in Ham’s F12 Medium (Corning) containing 2 mM L-glutamine, 100 U/mL penicillin, 100 μg/mL streptomycin, and 10% (vol/vol) fetal bovine serum (Gibco). CHO-K1 cells stably expressing full length MAdCAM-1 or MAdCAM-1-Δmucin by electroporation and selection by 0.2 mg/ml hygromycin (Amresco) as described (Yue et al., 2013).

### Flow Chamber Assay

Flow chamber assay was performed as described (Chen et al., 2003; Lu et al., 2016). To study integrin α4β7-mediated cell adhesion to recombinant proteins, a polystyrene Petri dish was coated with a 5 mm diameter, 20 μl spot of HS (100 μg/ml), MAdCAM-1 (25 μg/ml) or MAdCAM-1-Δmucin (100 μg/ml) in coating buffer (PBS, 10 mM NaHCO_3_, pH 9.0) for 1 h at 37°C, and then treated by 2% BSA in coating buffer for 1 h at 37°C to block non-specific binding sites. Otherwise, CHO-K1/MAdCAM-1 or CHO-K1/MAdCAM-1-Δmucin cells were seeded to a polystyrene Petri dish the day before the experiment, and washed by HBS (20 mM Hepes, pH 7.4) twice to clean up the culture medium. Then CHO-K1 cells were treated with HBS or 0.01 U/ml sialidase in HBS for 20 min at 37°C to remove cell surface anionic sialic acid of glycoconjugate. Integrin α4β7 expressing 293T cells were washed twice with HBS containing 5 mM EDTA and 0.5% BSA and diluted to 1 × 10^6^/ml in buffer A (HBS, 0.5% BSA) containing 1 mM Ca^2+^ + Mg^2+^. Then cells were infused in the chamber using a Harvard apparatus programmable syringe pump immediately. Cells were allowed to accumulate for 30 s at 0.3 dyn/cm^2^ and for 10 s at 0.4 dyn/cm^2^. Afterward, shear stress was increased every 10 s from 1 dyn/cm^2^ up to 32 dyn/cm^2^ in 2-fold increments. The rolling velocity at each shear stress was calculated from the average distance traveled by rolling cells in 3 s. A velocity of 1 μm/s corresponded to a movement of 1/2 cell diameter during the 3 s measurement interval. It was the minimum velocity required to define a cell adhesion behavior as rolling or firmly adherent. The number of cells remaining bound at the end of each 10 s interval was determined. Cells were pre-treated with murine IgG (10 μg/ml) or α4β7 blocking antibody Act-1 (10 μg/ml) for 10 min at 37°C before they were infused into the flow chamber.

### Flow Cytometry

Flow cytometry was performed as described (Lu et al., 2016). CHO-K1/MAdCAM-1 or CHO-K1/MAdCAM-1-Δmucin cells were stained with monoclonal antibody 8C1 against human MAdCAM-1 and then measured using FACSCelesta^TM^ (BD Biosciences). Data were analyzed using FlowJo 7.6.1 software.

### Fluorescence Resonance Energy Transfer (FRET)

For detecting the orientation of MAdCAM-1 ectodomain relative to cell membrane, CHO-K1/MAdCAM-1 or CHO-K1/MAdCAM-1-Δmucin cells were seeded on poly-L-Lysine (100 μg/ml) coated surface in HBS and incubated for 30 min at 37°C. Adherent cells were treated with HBS or 0.01 U/ml sialidase in HBS for 20 min at 37°C to remove cell surface anionic sialic acid of glycoconjugate. Then cells were fixed with 3.7% paraformaldehyde for 10 min at room temperature. Non-specific sites were blocked by incubation with 10% serum in HBS for 10 min at room temperature. 20 μg/ml Alexa Fluor 488-conjugated 8C1 Fab fragment was used to stain cells for 30 min at 37°C. Then cells were washed twice with HBS and labeled with 10 μM FM^TM^ 4-64FX (Invitrogen) for 1 min on ice. After one wash, cells were immediately mounted with Mowiol^®^ 4-88 (Polysciences) mounting solution under a coverslip. The slides were kept in dark and subjected to photobleach FRET acquisition by a confocal microscope (TCS SP8, Leica). FRET efficiency (E) was calculated as E = 1–(F_*donor*_(d)_*Pre*_/F_*donor*_(d)_*Post*_), where F_*donor*_(d)_*Pre*_ and F_*donor*_(d)_*Post*_ are the mean donor emission intensity of pre- and post-photobleaching.

### Quantification and Statistical Analysis

Statistical significance was determined by Student’s t test using Prism software (GraphPad, version 5.01). The resulting p values are indicated as follows: ns, not significant; ^∗^*p* < 0.05; ^∗∗^*p* < 0.01; ^∗∗∗^*p* < 0.001. Data represent the mean ± SEM of at least three independent experiments.

## Data Availability Statement

The original contributions presented in the study are included in the article/Supplementary Material, further inquiries can be directed to the corresponding author/s.

## Author Contributions

CL and JC conceptualized the project and designed the experiments. MY, YY, and YL performed the experiments. ZY, CL, and JC interpreted the results. CL drafted the manuscript. JC edited the manuscript. All authors contributed to the article and approved the submitted version.

## Conflict of Interest

The authors declare that the research was conducted in the absence of any commercial or financial relationships that could be construed as a potential conflict of interest.

## Supplementary Material

Supplementary Figure 1 MAdCAM-1 expression on CHO-K1/MAdCAM-1 and CHO-K1/MAdCAM-1-Δmucin cells. MAdCAM-1 expression on CHO-K1/MAdCAM-1 and CHO-K1/MAdCAM-1-Δmucin cells was determined by flow cytometry. Numbers within the panel showed the specific mean fluorescence intensities. Opened histogram: mock control.

Supplementary Movie 1 Adhesive behaviors of 293T-α4β7 cells on immobilized HS (100 μg/ml).

Supplementary Movie 2 Adhesive behaviors of 293T-α4β7 cells on immobilized MAdCAM-1 (25 μg/ml) substrates. Cells were pre-treated with murine IgG (10 μg/ml) for 10 min at 37°C.

Supplementary Movie 3 Adhesive behaviors of 293T-α4β7 cells on immobilized HS (100 μg/ml) and MAdCAM-1 (25 μg/ml) substrates. Cells were pre-treated with murine IgG (10 μg/ml) for 10 min at 37°C.

Supplementary Movie 4 Adhesive behaviors of 293T-α4β7 cells on immobilized MAdCAM-1 (25 μg/ml) substrates. Cells were pre-treated with α4β7 blocking antibody Act-1 (10 μg/ml) for 10 min at 37°C.

Supplementary Movie 5 Adhesive behaviors of 293T-α4β7 cells on immobilized HS (100 μg/ml) and MAdCAM-1 (25 μg/ml) substrates. Cells were pre-treated with α4β7 blocking antibody Act-1 (10 μg/ml) for 10 min at 37°C.
