# Supplementary figures and images for: Mucin-Like Domain of Mucosal Addressin Cell Adhesion Molecule-1 Facilitates Integrin α4β7-Mediated Cell Adhesion Through Electrostatic Repulsion
Source: Front Cell Dev Biol. 2020 Dec 14;8:603148. doi: 10.3389/fcell.2020.603148 (PMC7767916; doi:10.3389/fcell.2020.603148)

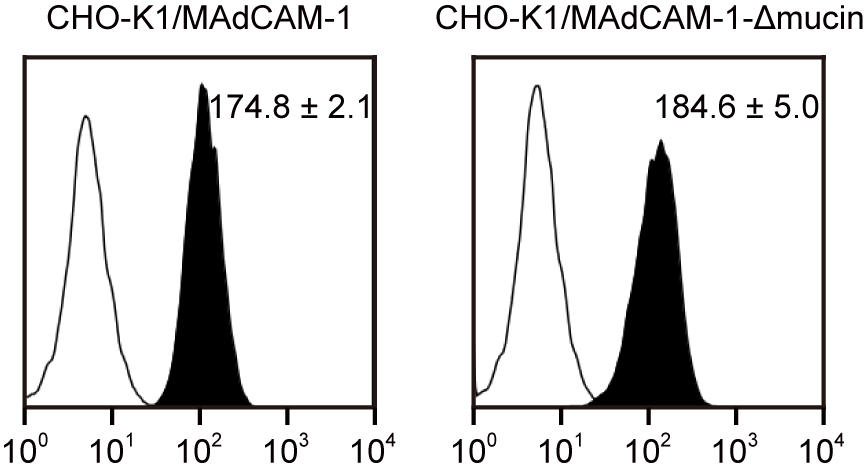

Supplement: Supplementary Figure 1 — MAdCAM-1 expression on CHO-K1/MAdCAM-1 and CHO-K1/MAdCAM-1-Δmucin cells. MAdCAM-1 expression on CHO-K1/MAdCAM-1 and CHO-K1/MAdCAM-1-Δmucin cells was determined by flow cytometry. Numbers within the panel showed the specific mean fluorescence intensities. Opened histogram: mock control. [file Image_1.TIF]
